# Maternal Outcomes Among Pregnant Women Diagnosed With COVID-19

**DOI:** 10.7759/cureus.33887

**Published:** 2023-01-17

**Authors:** Ahlam Al-Zahrani, Latifah Alanazi, Hala Thabet, Faiz Alenezi

**Affiliations:** 1 Maternity and Child Department, Nursing Faculty, King Abdulaziz University, Jeddah, SAU; 2 Nursing, Maternal and Child Hospital, Ministry of Health, Arar, SAU; 3 Woman's Health & Midwifery Nursing Department, Nursing Faculty, Mansoura University, Mansoura, EGY; 4 Pharmacy, Ministry of Health, Arar, SAU

**Keywords:** kingdom of saudi arabia, pregnant women, covid-19 in pregnancy, coronavirus disease, maternal outcomes

## Abstract

Background: COVID-19 has the potential development of negative maternal outcomes for pregnant women. The risk of contracting COVID-19 is high as pregnancy alters the maternal immune system. Therefore, this study aims to assess maternal outcomes among pregnant women with COVID-19 in the Kingdom of Saudi Arabia.

Methods: A retrospective study was conducted in three hospitals during the pandemic over four months, from the beginning of December 2019 until the end of March 2020. Data was collected using a structured questionnaire filled by the researcher using computers from the medical records of three hospitals. The sampling was all confirmed cases of pregnant women who delivered while being positive for COVID-19.

Results: This study has identified a total of 82 pregnant women with confirmed COVID-19 infection over the study period, with ages ranging from 18 to >40 years. The majority of the pregnant women (84.1%) were symptomatic, with fever (48.8%) being the most frequent COVID-19 symptom, followed by cough (42.7%) and shortness of breath (41.5%). Some women (46.3%) had a spontaneous normal vaginal delivery, and 50.2% had a cesarean delivery. The most common adverse pregnancy outcome was premature delivery (36.5%), followed by fetal distress (20.7%), preeclampsia (2.4%), eclampsia (1.2%), and diabetic ketoacidosis (1.2%), as well as the death of three pregnant women.

Conclusion: This study found that infected mothers faced various risks of maternal adverse outcomes. The majority of the pregnant women experienced mild to moderate illness symptoms and were delivered within 14 days of the onset of COVID-19 symptoms. Healthcare providers should provide more attention to pregnant women diagnosed with COVID-19.

## Introduction

COVID-19 is a critical risk for pregnant women across the globe and causes different adverse maternal outcomes. It is caused by a severe acute respiratory syndrome called coronavirus-2 (SARS-CoV-2). Pregnant women are considered to be at significant risk of contracting viral respiratory infections and developing symptoms of severe pneumonia because of the changes in their physiological characteristics, such as their immunological and cardiopulmonary systems, which occur during pregnancy [1].

COVID-19 has the potential development of negative maternal outcomes after the disease affects the respiratory organs of pregnant mothers. The risk of contracting COVID-19 disease is high as pregnancy alters the maternal immune system. Consequently, the global pandemic is a constant threat to fetal development and the health of pregnant mothers [2]. It was reported that the growing COVID-19-related knowledge of virology, clinical, genetic, and epidemiology has prompted the consideration of risks faced by pregnant mothers across the globe [2]. The researchers have argued that the immunocompromised nature of pregnant women implies potential complications arising from COVID-19-related infections [3].

According to the Ministry of Health (MOH) in the Kingdom of Saudi Arabia (KSA), around 600,000 cases were infected with COVID-19 [4]. The KSA government took the threat of COVID-19 seriously and immediately by taking numerous measures early on to slow down the spread of the COVID-19 virus [4]. The KSA began implementing strong and strict social distancing measures, which included everything from the suspension or cancellation of mass gatherings in religious, entertainment, and sporting events, such as the Umrah and pilgrimage, to the temporary closure of educational institutions and mosques, as well as the postponement of all gatherings and the imposition of a curfew as well as a total ban within all the KSA cities to prevent cases from worsening and becoming difficult to control [4]. The Saudi Society of Maternal-Fetal Medicine (SSMFM) formed a task force comprising the Maternal-Fetal Medicine (MFM) experts to review available evidence regarding pregnancy and COVID-19 to provide safe care for pregnant women in the KSA by exploring recent evidence that may help in prevent COVID-19 transmission and provide management recommendations for suspected/confirmed COVID-19 patients [5].

The International Society of Infectious Disease in Obstetrics and Gynecology (ISIDOG) provided critical guidelines to shape the diagnosis and management of pregnant women with COVID-19 [6]. The potential effect of viral infection on maternal health necessitates special care to improve mothers’ survival rate [7]. The protection of pregnant mothers necessitates the input of healthcare professionals and patients, especially in isolation wards [8]. However, the lack of extensive research on the maternal outcomes of pregnant women with COVID-19 in the KSA laid a weak foundation for the literature required to enhance maternal care; hence, the current research aims to assess the maternal outcomes among pregnant women diagnosed with COVID-19 in the KSA.

## Materials and methods

Study design

This study used a quantitative retrospective design to assess the maternal outcomes of childbearing women diagnosed with COVID-19. The research process entailed an examination of clinical records and laboratory results on maternal outcomes. This design involved looking backward and examining the exposure of childbearing women to COVID-19 regarding the maternal outcomes established from medical records. In addition, this design assisted in developing ideas and potential associations between the outcomes and the risks [9]. The design allowed for the examination of administrative datasets or patient charts to investigate and describe a population over time.

Study setting

Data for the present study were collected from three hospitals in the KSA. Two were located in Jeddah, and one in Arar city. These hospitals were included based on the approval for data collection. These hospitals are East Jeddah General Hospital (EJGH) and King Abdullah Medical Complex (KAMC) in Jeddah city and Maternity and Children Hospital (MCH) in Arar city. All these hospitals are big in capacity and provide free care for pregnant women. 

Study sample

This study is a retrospective study that included all cases available in the medical records for childbearing women who were infected with COVID-19. The inclusion criteria included full- and pre-term birth and who had delivered in the three selected hospitals. The data was collected from the medical records of the three hospitals. All medical records for childbearing women with positive COVID-19 results were included in the sample. As a result, the sample comprises 82 cases.

Study tool

For this study, a structured questionnaire was developed by the researchers guided by the literature and was reviewed by three faculty members specialized in obstetrics and gynecology in the Faculty of Nursing at King Abdulaziz University (KAU). The structured questionnaire facilitated the assessment of the maternal outcomes among participants with COVID-19 through medical records. The questionnaire comprised seven parts that queried socio-demographic data: obstetrics, medical history, surgical history, maternal assessment, laboratory findings, clinical information, and maternal outcomes. The medical records were visited in advance before undertaking the main study to foster the adjustment of the questionnaire.

Data collection

The collection of data was conducted from the medical records over four months, from the beginning of December 2019 until the end of March 2020. Data was collected from the available medical records in the three hospitals using a structured questionnaire, which was used as a checklist filled by the researcher. All medical records for each woman who delivered and was diagnosed with COVID-19 were carefully searched and reviewed. The questionnaire was filled out accordingly for each sample, focusing on the details that addressed the research questions. 

Ethical considerations

Ethical approval of the research was first sought from the committee of the Nursing Faculty at KAU in Jeddah city (NREC Serial No: Ref No 1M. 11). Another ethical approval was sought from MOH to provide access to the three hospitals for data collection (H-09-A-51). The three hospitals offered access to the medical records of the women who were delivered with confirmed COVID-19. The retrospective cohort study aimed to use the medical records of the women focusing on details that address the research questions.

Statistical analysis

Statistical analysis was done using IBM Corp. Released 2017. IBM SPSS Statistics for Windows, Version 25.0. Armonk, NY: IBM Corp. As applicable, all continuous variables were reported as a median and interquartile range (IQR). The frequencies and percentages were used to represent all categorical variables. Additionally, a Pearson’s correlation test was undertaken to establish the relationship between some sociodemographic data and various maternal outcomes.

## Results

Participant’s demographic data

This study has identified 82 pregnant women who had been diagnosed with confirmed COVID-19 infection from three hospitals. Table 1 shows that more than half (61.0%) of the participants in this study are 26-35 years old, 15.9% of them are 36-40 years old, and 12.1% are 18-25 years old. It was found that 46.3% of the study participants have a university degree, 39.0% have secondary school education, and 14.6% are less educated than secondary school. Moreover, 78.0% of the participants are housewives, while 19.5% are working women. There are no participants in this study who smoke. In addition, more than half (54.9%) of the participants are obese, more than one-quarter (29.3%) are overweight, and the rest (15.8%) have normal body mass index (BMI).

**Table 1:** Participant’s demographic data

Demographic data	Number			Percentage
Age group (years)				
18–25	10		12.1	
26–35	50		61.0	
36–40	13		15.9	
>40	9		11.0	
Education level				
Below secondary		12	14.6	
Secondary		32	39.0	
University		38	46.3	
Occupation				
Housewife		64		78.0
Student		2		2.4
Working		16		19.5
Smoking				
No		82		100.0
Body mass index				
18.5 to <25 (normal)		13		15.8
25.0 to <30 (overweight)		24		29.3
30.0 or higher (obese)		45		54.9
Total		82		100.0

Participant’s medical and surgical history

Table 2 represents the frequency distribution regarding the medical and surgical history of the study participants. It was illustrated that 75.6% of the participants have no medical history, 7.3% have bronchial asthma, 7.3% have diabetes mellitus, 4.9% have hypothyroidism, and 1.2% have hypothyroidism and bronchial asthma. In addition, 1.2% have anemia, and 1.2% have chest allergies. Moreover, 69.5% have no surgical history. More than two-thirds (69.5%) of the study participants do not have a surgical history of diseases, and more than one-quarter (29.3%) have undergone a cesarean section.

**Table 2:** Medical and surgical history of the study participants

Medical and surgical history	Number N=82	Percentage
Medical history		
None	62	75.6
Diabetic mellitus	6	7.3
Hypothyroidism	4	4.9
Bronchial asthma	6	7.3
Hypothyroidism and bronchial asthma	1	1.2
Chest allergy	1	1.2
Anemia	1	1.2
Hypertension and diabetic	1	1.2
Surgical history		
None	57	69.5
Appendectomy	1	1.2
Caesarean section	24	29.3

Obstetric history of the study participants

Table 3 summarizes the frequency distribution regarding the history of the study participants. The table revealed that more than three-thirds (78.0%) of the participants are multigravida, and only more than one-fifth (22.0%) are primigravida. In addition, more than half (61.0%) of the participants had 1-3 deliveries, 13.4% had 4-6 deliveries, and 3.7% had 7-9 deliveries. Moreover, the majority (80.5%) did not have abortions; 14.6% had 1-2 abortions, while the rest (4.9%) had 3-4 abortions.

**Table 3:** Obstetric history of the study participants

Obstetric history	Number (N=82)	Percentage
Gravida		
Primigravida	18	22.0
Multigravida	64	78.0
Para		
0	18	22.0
1–3	50	61.0
4–6	11	13.4
7–9	3	3.7
Abortion		
0	66	80.5
1–2	12	14.6
3–4	4	4.9
Previous obstetric complications		
None	73	89.0
Preeclampsia	3	3.7
Gestational diabetes	5	6.1
Deep venous thrombosis	1	1.2
Previous fetal complications		
None	80	97.6
Preterm	2	2.4

It was also revealed that the majority of the participants (89.0%) did not have previous obstetric complications, 6.1% had gestational diabetes, and 3.7% had preeclampsia. Moreover, regarding previous fetal complications, 97.6% of the participants did not have fetal complications, while 2.4% had complications.

Maternal assessment

Table 4 revealed that about two-fifths (39.0%) of the study participant’s gestational age is between 38 and <40 weeks: the gestational age of 20.7% is between 37 and <38 weeks, while the gestational age of 19.5% is less than 35 weeks. In addition, the majority (85.4%) of the study participants have a normal temperature ranging from 35.8 to 37.4°C, while the rest of them (14.6%) have an abnormal temperature (>37.4°C).

**Table 4:** General maternal assessment for the study participants

General maternal assessment	Number (N=82)		Percentage
Gestational age (weeks)			
<35	16		19.5
35‎–<36	2		2.4
36–<37	12		14.6
37–<38	17		20.7
38–<40	32		39.0
≥40	3		3.7
Temperature (°C)			
35.8–37.4	70		85.4
>37.4	12		14.6
Places of admission to the delivery room			
Emergency	70		85.3
Isolation	5		6.1
Intensive care unit	7		8.5
Cervical status up on admission			
Closed	40		48.8
1–3	19		23.2
4–7	18		22.0
8–10	5		6.1
Condition of membrane			
Intact	59		72.0
ARM	3		3.7
PRM	20		24.4

In addition, Table 4 shows that the majority (85.3%) of the participants were admitted through the emergency room, while the place of admission to the delivery room of 8.5% of the participants was the intensive care unit. In addition, the cervical status upon admission for about half (48.8%) of the participants was closed, more than one-fifth (23.2%) had cervical dilatation at 1-3 cm (latent phase), while 22.0% had cervical dilatation at 4-7 cm (active phase). Regarding the condition of the membrane, more than two-thirds (72.0%) of the participants had intact membranes; 24.4% had premature rupture of membrane (PRM), while the rest (3.7%) had artificial rupture of membrane (ARM).

Delivery assessment

Table 5 represents the delivery assessment of participants; it shows that the majority (42.7%) of the participants had spontaneous onset of labor, 40.2% had a cesarean section before labor, and 15.9% had induced onset of labor. In addition, 19.5% had vaginal watery discharge, 24.4% had uterine contractions, 15.9% had desaturation, and 12.2% had decreased or no fetal movement.

**Table 5:** Delivery assessment

Delivery assessment	Number (N=82)			Percentage	
Onset of labor					
Unknown	1			1.2	
Spontaneous	35			42.7	
Induced	13			15.9	
Caesarean section before labor	33			40.2	
Symptoms upon admission					
None	17			20.7	
Vaginal watery discharge	16			19.5	
Vaginal bleeding	2			2.4	
Desaturation	13			15.9	
Pain	4			4.9	
Decreased or no fetal movement	10			12.2	
Uterine contractions	20			24.4	
Fetal heart rate					
No fetal heart rate		4			4.9
<100 b/m		2			2.4
110 to <130 b/m		21			25.6
130 to <160 b/m		53			64.6
>160 b/m		2			2.4
Treatment during hospitalization					
None		57	69.5		
Induction of labor		15	18.3		
Blood transfusion		5	6.1		
Other		5	6.1		

Regarding fetal heart rate (FHR), 64.6% had an FHR of 130 to <160 b/m, and 25.6% had an FHR of 110 to <130 b/m. Regarding treatment during hospitalization, 18.3% had induction of labor, and 6.1% had a blood transfusion.

COVID-19 results and symptoms for the study participants

Table 6 shows that a majority (91.5%) of the study participants had positive COVID-19 test results before admission, while 8.5% had it upon admission. In addition, a majority (84.1%) of the participants had symptoms related to COVID-19, while 15.9% were asymptomatic. Regarding symptoms of COVID-19, about half (48.8%) of the participants had a fever, 4.9% had a sore throat, 8.5% had a runny nose, 42.7% had a cough, and 41.5% had shortness of breath. Table 6 also shows that 6.1% of the participants had nausea, 13.4% had vomiting, 14.6% had a headache, 18.3% had muscle pain, 18.3% had joint pain, and 11.0% had diarrhea.

**Table 6:** COVID-19 test results and symptoms

COVID-19 test results and symptoms	Number N=82	Percentage
Positive COVID-19 test results		
Prior admission	75	91.5
Upon admission	7	8.5
Asymptomatic		
Yes	13	15.9
No	69	84.1
Fever		
Yes	40	48.8
No	42	51.3
Sore throat		
Yes	4	4.9
No	78	95.2
Runny nose		
Yes	7	8.5
No	69	91.5
Cough		
Yes	35	42.7
No	46	57.4
Shortness of breath		
Yes	34	41.5
No	48	57.9
Nausea		
Yes	5	6.1
No	77	93.9
Vomiting		
Yes	11	13.4
No	71	86.6
Headache		
Yes	12	14.6
No	70	85.4
Muscle pain		
Yes	15	18.3
No	67	81.7
Joint pain		
Yes	15	18.3
No	67	81.7
Diarrhea		
Yes	9	11.0
No	73	89.0

Maternal outcomes

Table 7 shows that 46.3% of the participants had a spontaneous vaginal delivery and 40.2% had an unplanned cesarean, and 11.2% had a scheduled cesarean. The reasons for caesarean section included fetal distress (20.7%), maternal fatigue (4.9%), and other causes (25.6%). In addition, 95.2% did not have complications during pregnancy, 2.4% had preeclampsia, 1.2% had eclampsia, and 1.2% had diabetic ketoacidosis. Moreover, 92.7% of the participants did not have complications after delivery, while 3.7% had excessive bleeding.

**Table 7:** Maternal outcomes for the study participants

Maternal outcomes	Number N=82	Percentage
Mode of delivery		
Spontaneous normal vaginal delivery	38	46.3
Assisted vaginal delivery (ventouse, forceps)	1	1.2
Scheduled caesarean	9	11.0
Unplanned caesarean	34	41.4
Reason for caesarean section		
Fetal distress	17	20.7
Maternal fatigue	4	4.9
Other	21	25.6
Current abnormal pregnancy		
None	78	95.2
Preeclampsia	2	2.4
Eclampsia	1	1.2
Diabetic ketoacidosis	1	1.2
Complication after delivery		
None	76	92.7
Excessive bleeding	3	3.7
Other	3	3.7
Complication of COVID-19		
Nnoe	57	69.5
Intensive care unit admission	12	14.6
Intubation	10	12.2
Maternal death	3	3.7
Length of hospitalization		
≤3 days	37	45.1
4–6 days	25	30.5
>6 days	20	24.4
Delivery within 14 days from onset of symptom		
Yes	70	85.4
No	12	14.6

Regarding the complications of COVID-19, 69.5% of the participants did not have complications, 14.6% have been admitted to the intensive care unit admission, 12.2% had intubation, and 3.7% had maternal death. In addition, 96.3% of the participants have been discharged from the hospital. Moreover, 45.1% of the participants have been admitted to the hospital for three days or less, 30.5% have been admitted for 4-6 days, and 24.4% have been admitted for more than six days.

Association between participants' age and maternal outcomes

According to the results presented in Table 8, there is no significant association between maternal age and maternal outcomes, including mode of delivery, complication after delivery, complication of COVID-19, clinical outcome, and length of hospitalization (p>0.05).

**Table 8:** Association between age and maternal outcomes

Maternal outcomes	18–25	26–35	36–40	>40 years	Total	Chi-square	p-value
Mode of delivery							
Spontaneous normal vaginal	6 (60.0)	22 (44.0)	9 (69.2)	1 (11.1)	38 (46.3)	11.238	0.509
Assisted vaginal delivery	0 (0.0)	1 (2.0)	0 (0.0)	0 (0.0)	1 (1.2)		
Scheduled cesarean	1 (10.0)	7 (14.0)	0 (0.0)	1 (11.1)	9 (11.0)		
Unplanned caesarean	3 (30.0)	20 (40.0)	4 (30.8)	7 (77.8)	34 (41.4)		
Complication after delivery							
None	9 (90.0)	46 (92.0)	12 (92.3)	9 (100.0)	76 (92.7)	4.365	0.627
Excessive bleeding	1 (10.0)	1 (2.0)	1 (7.7)	0 (0.0)	3 (3.7)		
Other	0 (0.0)	3 (6.0)	0 (0.0)	0 (0.0)	3 (3.7)		
Complication of COVID-19							
None	10 (100.0)	43 (86.0)	11 (84.6)	6 (66.7)	70 (85.4)	10.677	0.299
Intensive care unit admission	0 (0.0)	1 (2.0)	1 (7.7)	2 (22.2)	4 (4.9)		
Intubation	0 (0.0)	3 (6.0)	1 (7.7)	1 (11.1)	5 (6.1)		
Maternal death	0 (0.0)	3 (6.0)	0 (0.0)	0 (0.0)	3 (3.7)		
Clinical outcome							
Discharge from hospital	10 (100.0)	47 (94.0)	13 (100.0)	9 (100.0)	79 (96.3)	1.993	0.574
Died	0 (0.0)	3 (6.0)	0 (0.0)	0 (0.0)	3 (7.3)		
Length of hospitalization							
≤3 days	7 (70.0)	23 (46.0)	5 (38.5)	2 (22.2)	37 (45.1)	8.364	0.213
4–6 days	2 (20.0)	17 (34.0)	4 (30.8)	2 (22.2)	25 (30.5)		
>6 days	1 (10.0)	10 (20.0)	4 (30.8)	5 (55.6)	20 (24.4)		

Association between occupation and maternal outcomes

Table 9 shows that there is a significant association between occupation and maternal outcome (p<0.05) in favor of housewives. On the other hand, there is no significant association between occupation and other maternal outcomes, including mode of delivery, a complication of COVID-19, clinical outcome, and length of hospitalization (p>0.05).

**Table 9:** Association between participant's occupation and maternal outcomes

Maternal outcomes	Housewife	Student	Working	Total	Chi-square	p-value
Mode of delivery						
Spontaneous normal vaginal	28 (43.8)	2 (100.0)	8 (50.0)	38 (46.3)	7.363	0.498
Assisted vaginal delivery	1 (1.6)	0 (0.0)	0 (0.0)	1 (1.2)		
Scheduled cesarean	7 (10.9)	0 (0.0)	2 (12.5)	9 (11.0)		
Unplanned caesarean	28 (43.8)	0 (0.0)	6 (37.4)	34 (41.4)		
Abnormal delivery						
None	64 (100.0)	2 (100.0)	15 (93.8)	81 (98.8)	4.176	0.124
Other	0 (0.0)	0 (0.0)	1 (6.2)	1 (1.2)		
Complication after delivery						
None	60 (93.8)	1 (50.0)	15 (93.8)	76 (92.7)	14.049	0.007
Excessive bleeding	1 (1.6)	1 (50.0)	1 (6.2)	3 (3.7)		
Other	3 (4.7)	0 (0.0)	0 (0.0)	3 (3.7)		
Complication of COVID-19						
None	55 (85.9)	2 (100.0)	13 (81.2)	70 (85.4)	2.498	0.869
Intensive care unit admission	3 (4.7)	0 (0.0)	1 (6.2)	4 (4.9)		
Intubation	3 (4.7)	0 (0.0)	2 (12.5)	5 (6.1)		
Maternal death	3 (4.7)	0 (0.0)	0 (0.0)	3 (3.7)		
Clinical outcome						
Discharge from hospital	61 (95.3)	2 (100.0)	16 (100.0)	79 (96.3)	0.876	0.645
Died	3 (4.7)	0 (0.0)	0 (0.0)	3 (3.7)		
Clinical outcomes						
≤3 days	31 (48.4)	1 (50.0)	5 (31.2)	37 (45.1)	2.294	0.682
4–6 days	18 (28.1)	1 (50.0)	6 (37.5)	25 (30.5)		
>6 days	15 (23.4)	0 (0.0)	5 (31.2)	20 (24.4)		

## Discussion

This study aimed to assess maternal outcomes among childbearing women diagnosed with COVID-19 in the KSA. This study retrospectively includes all pregnant women with COVID-19 infection in three hospitals. The demographic characteristics of the participants revealed that more than half of the participants in this study are 26-35 years old. Also, more than three-thirds of the participants were multigravida, while more than one-fifth were primigravidas. These findings are similar to other studies [10-12]. These studies demonstrated that women with different demographic characteristics are susceptible to COVID-19 infections during and after their pregnancy. Therefore, it can be concluded that COVID-19 necessitates urgent attention irrespective of the different demographic characteristics of the pregnant women.

The results of this study reveal that pregnant women diagnosed with positive COVID-19 have both symptomatic and asymptomatic cases. It shows that the majority of pregnant women with COVID-19 infection were symptomatic. However, the most common symptoms for the symptomatic participants registered shortness of breath, coughing, diarrhea, fatigue, and fever, which is congruent with most previous research [10,13-18].

The severity of COVID-19 in this study was mild for the majority of the study participants, and some of them experienced severe or critical illness, which is similar to the findings found in other studies [16,18]. It was reported that pregnant women with COVID-19 pneumonia had a milder illness and a faster recovery [17]. They found that pregnant women who experienced milder symptoms are younger. It is worth mentioning that these study findings revealed that all participants were aged between 18 and 44 years, and the majority had a mild illness. It can argue that the immune system weakens with older age, but in younger people, the immune systems are likely to be strong enough to protect pregnant women from any viral disease, including COVID-19.

The current findings demonstrate that the majority of participants had delivered within 14 days of the onset of COVID-19 symptoms. This implies that the severity of COVID-19 has an impact on pregnancy outcomes, as evidenced that more than one-third of the study participants had a preterm birth. Moreover, all pregnant women who experienced severe or critical illness in this study had delivered premature babies. These findings are in line with other studies, which reported that premature births happened when the mothers had severe or critical conditions [1,17,19]. Infection with COVID-19 at the early phase of pregnancy, as well as the severity of the illness, might trigger contractions during pregnancy that cause the cervix to open before the 37th week of pregnancy and lead to preterm birth. These data imply that the severity of COVID-19 is a risk factor that can affect the outcomes of both the mother and the fetus. Prematurity can cause health difficulties for babies, and they may need to stay in the hospital for an extended period. This also can have long-term cost consequences.

Many studies have reported that premature birth, fetal distress, preeclampsia, placenta abruption, gestational diabetes, hypertension, fetal hypoxia, stillbirth, abortion, and maternal death were among the most common unfavorable pregnancy outcomes [10,13,14,18,20,21]. It is worth mentioning that fetal distress, maternal fatigue, preeclampsia, and eclampsia were the most common reasons for preterm delivery among women with COVID-19 in this study, which are in parallel with other studies [11]. This recommends that pregnant women should practice diligently to avoid contracting an infection during the first and second trimester; it seems that the severity of COVID-19 can influence contraction and lead to preterm birth.

The study revealed that more than half of the participants are obese, and more than one-quarter are overweight. It was also found that the majority of study participants who had severe symptoms were admitted to ICU and required mechanical ventilation, and their BMI was more than 35. This finding concurs with other studies [11,14]. They reported that women who were overweight at their initial antenatal appointment and were diagnosed with COVID-19 were at the greatest risk for maternal morbidity and mortality. It can, however, take into account the prevalence of obesity in the KSA and were 24.7% in a recent national-level survey [22]. It can be concluded that obesity is a predictor of complications registered by the mothers with COVID-19.

The majority of participants in this study had positive COVID-19 symptoms prior to admission, and only a few were diagnosed with COVID-19 during hospitalization. Proper diagnosis is imperative for the women, as it helps with monitoring pregnancy and potential complications. However, it was argued that the clinical presentations and symptomatic COVID-19 status determines the intervention to prevent possible fetal distress, unplanned CS, and/or vertical transmission [23]. The COVID-19 tests and early detection are critical for better maternal, fetal, and neonatal outcomes while enabling the medical teams to take proper precautions to safeguard women against severity of complications.

The results of this study show that the CS, whether booked or unbooked, was the most common route of delivery in the majority of cases. The study results were also in line with various studies [11,24-27]. However, in this study, the major reason for CS is because of fetal distress, which is similar to other studies that reported that the majority of caesarean deliveries were performed for reasons other than maternal SARS-CoV-2 infection, such as fetal compromise [28]. It can therefore be argued that the delivery through CS is needed in some cases to prevent further gestation or related risks for mothers with COVID-19.

The results of this study revealed varying levels of hospitalization depending on the severity of the disease; almost half of the mothers were hospitalized before the onset of labor because of obstetric illness as well as COVID-19 infection. These findings concord with the findings of other studies [3,29]. Therefore, it can be argued that the essence of hospitalization is to manage the potential severity of the condition to enhance the quality of health outcomes of the mother or fetus, irrespective of COVID-19 status.

The findings of this study revealed that study participants who experienced critical complications of COVID-19 led to respiratory failure, renal failure, sepsis, and maternal deaths. These results are in line with other studies, which detailed cases of COVID-19-related severe illness in pregnant women and maternal death [14,30-32]. Therefore, it can be argued that COVID-19 during pregnancy can be an indicator to increase the possibility of complications for maternal outcomes. A woman’s death during childbearing is tragic. Maternal death must be considered a completely avoidable tragedy [33]. Praise to God, there were few deaths in this study, which may be attribute to the KSA’s advanced healthcare, as well as to the early precautions taken by the women and the Saudi government in confronting the pandemic.

The study was conducted at three hospitals within a limited period, which limited the number of COVID-19 cases included (n=82). A small sample size reduced the generalizability of the research findings in pregnant women with COVID-19 with different maternal outcomes and healthcare institutions. Therefore, further research is required with large samples and diverse geographical areas to establish the generalizability of the study. 

## Conclusions

This is a retrospective study which took place in three hospitals in the KSA. This study confirms the findings that have been reported in other national and international studies that aimed to assess the impact of COVID-19 on the maternal outcome. Infected mothers faced various risks of maternal adverse outcomes such as preterm birth, cesarean birth, ICU admission, intubation, post-partum hemorrhage, transfusion, fetal loss, and maternal death. Healthcare providers should provide more attention to pregnant women diagnosed with COVID-19.
